# Supplementary material for: Thermodynamics Beyond Molecules: Statistical Thermodynamics of Probability Distributions
Source: Entropy (Basel). 2019 Sep 13;21(9):890. doi: 10.3390/e21090890 (PMC7515426; doi:10.3390/e21090890)
Supplement: Supplementary file 1 [file entropy-21-00890-s001.pdf]

SUPPLEMENTARY INFORMATION

DERIVATIONS

This document gives derivations of results that appear in the paper. All equation numbers not prefixed by "SI" refer to the manuscript.

Homogeneous Bias (Equation 14)

Homogeneity allows us to express $\log W$ as an integral over the variational derivatives $\log w(x;h)$,

$$\boxed{\log W[h] = \int h(x)\frac{\delta \log W[h]}{\delta h}dx = \int h(x)\log w(x;h)dx.} \tag{SI.1}$$

This is Eq. (14) in the text. We also have

$$\int h(x)\delta \log w(x;h)dx = 0, \tag{SI.2}$$

or equivalently,

$$\boxed{\int h(x)\frac{\partial \log w(x;h)}{\partial t}dx = 0,} \tag{SI.3}$$

where t is any parameter other than x on which h may depend (for example, $\bar{x}$, β, etc., or any function of these variables). In the special but important case that $\log W[h]$ is linear functional of h, i.e.,

$$\log W[h] = \int h(x)a(x)dx, \tag{SI.4}$$

where $a(x)$ is a fixed function of x, Eq. (SI.1) is satisfied with $\log w(x;h) = a(x)$, and Eq. (SI.3) is satisfied trivially, since in this case $\delta a(x)/\delta h = 0$ ($a(x)$ does not depend on h).

Equations (SI.1) and (SI.3) are the equivalents of the following two results for homogeneous functions $f(x_1, x_2\cdots)$ of degree 1 with respect to all x_i, extended to functionals:

$$f(x_1, x_2\cdots) = \sum_i x_i\frac{\partial f_i}{\partial x_1}, \tag{SI.5}$$

$$0 = \sum_i x_i d\left(\frac{\partial f_i}{\partial x_1}\right), \tag{SI.6}$$

Equation (SI.1) is used throughout the paper. Equation (SI.3) is used in the derivation of Eq. (25) later in this Supplement.

Most Probable Distribution in Biased Sampling (Equation 20)

We maximize the generic probability functional (Eq. (16) in the paper)

$$\log \varrho = -\int h(x) \log \frac{h(x)}{w(x;h)h_0(x)} dx - \log r, \tag{SI.7}$$

with respect to h under the normalization constraint

$$\int h(x) dx = 1. \tag{SI.8}$$

Using the Lagrange multiplier λ_0, the equivalent unconstrained maximization problem is

$$\max_h \left\{ -\int h(x) \log \frac{h(x)}{w(x;h)h_0(x)} dx - \lambda_0 \left(\int h(x) dx - 1 \right) - \log r \right\}, \tag{SI.9}$$

with q, λ_0 and r fixed. We set the variational derivative at $h = h_*$ equal to zero,

$$0 = -\log h^*(x) - 1 + \log w(x; h^*) + \log h_0(x) - \lambda_0, \tag{SI.10}$$

and solve for h^* to obtain

$$h^*(x) = \frac{w(x;h^*)h_0(x)}{e^{1+\lambda_0}} = \frac{w(x;h^*)h_0(x)}{\alpha}, \tag{SI.11}$$

with $\alpha = e^{1+\lambda_0}$. To evaluate r we apply the condition $\varrho[h^*|W, h_0] = 1$. Noting that

$$\frac{h^*(x)}{w(x;h^*)h_0(x)} = \frac{1}{\alpha}$$

we have:

$$0 = -\int h^*(x) \frac{h^*(x)}{w(x;h^*)h_0(x)} dx - \log r = \int h^*(x) \log \alpha \, dx - \log r = \log \frac{\alpha}{r},$$

and finally, $\alpha = r$. The most probable distribution is

$$\boxed{h^*(x) = \frac{w(x;h^*)h_0(x)}{r}.} \tag{SI.12}$$

This is Eq. (20) in the text.

Results in Canonical Space

Canonical Probability Functional (Equation 22)

We obtain the canonical functional by setting $h_0(x) = \beta e^{-\beta x}$ in Eq. (SI.7):

$$\begin{aligned} \log \varrho[h|W,\beta] &= -\int h(x) \log \frac{h(x)}{w(x;h)} dx + \int h(x) \log \beta e^{-\beta x} dx - \log r \\ &= -\int h(x) \log \frac{h(x)}{w(x;h)} dx - \beta \bar{x} - \log(r/\beta), \end{aligned} \tag{SI.13}$$

where $\bar{x}$ is the mean of h. We define $q = r/\beta$ and write the canonical functional as

$$\boxed{\log \varrho[h|W,\beta] = -\int h(x)\log\frac{h(x)}{w(x;h)}dx - \beta\bar{x} - \log q.} \tag{SI.14}$$

This is Eq. (22) in the text.

Most Probable Distribution in Canonical Space (Equation 24)

The canonical functional in Eq. (SI.14) is a special case of the generic functional in Eq. (SI.7) with $h_0 = \beta e^{-\beta x}$ and $q = r/\beta$. The most probable distribution of the generic probability functional is given in Eq. (SI.12); accordingly, the most probable distribution in the canonical space is obtained from that equation with $h_0(x) = \beta e^{-\beta x}$ and $r = q\beta$:

$$h^*(x) = w(x;h^*)\frac{\beta e^{-\beta x}}{\beta q}, \tag{SI.15}$$

or

$$\boxed{h^*(x) = w(x;h^*)\frac{e^{-\beta x}}{q},} \tag{SI.16}$$

which is Eq. (24) in the text.

The q-β-$\bar{x}$ Relationship (Equation 25)

We write Eq. (24) as

$$q = \int w(x;h^*)e^{-\beta x}dx$$

and take the derivative $d(\log q)/d\beta$:

$$\frac{d\log q}{d\beta} = -\underbrace{\int xw(x;h^*)\frac{e^{-\beta x}}{q}dx}_{\bar{x}} + \int\frac{\partial w(x;h^*)}{\partial\beta}\frac{e^{-\beta x}}{q}dx = -\bar{x} + \underbrace{\int\frac{\partial\log w(x;h^*)}{\partial\beta}h^*(x)dx}_{=0} = -\bar{x}. \tag{SI.17}$$

The last integral is identically equal to zero by virtue of Eq. (SI.3). The final result is

$$\boxed{\frac{d\log q}{d\beta} = -\bar{x},} \tag{SI.18}$$

which is Eq. (25) in the text.

Results in Microcanonical Space

Microcanonical Probability Functional (Equation 27)

The microcanonical functional in the continuous limit is

$$\log \varrho[h|h_0, \bar{x}] = -\int h(x) \log \frac{h(x)}{w(x;h) h_0(x)} dx - \log r', \tag{SI.19}$$

with r' such that normalization is satisfied. Setting $h_0 = e^{-x/\bar{x}}/\bar{x}$ we obtain

$$\begin{aligned} \log \varrho[h|h_0, \bar{x}] &= -\int h(x) \log \frac{h(x)}{w(x;h)} dx + \int h(x) \log \left(\frac{e^{-x/\bar{x}}}{\bar{x}} \right) - \log r' \\ &= -\int h(x) \log \frac{h(x)}{w(x;h)} dx - 1 - \log \bar{x} - \log r'. \end{aligned} \tag{SI.20}$$

Setting $\log \omega = -1 - \log \bar{x} - \log r'$ we obtain

$$\boxed{\log \varrho[h|W, \bar{x}] = -\int h(x) \log \frac{h(x)}{w(x;h)} dx - \log \omega,} \tag{SI.21}$$

which is Eq. (27) in the text.

Most Probable Distribution in Microcanonical Space (Equation 24)

We now show that that the distribution that maximizes the microcanonical functional is given by the same distribution as in the canonical case (Eq. 24 of the manuscript). We maximize the microcanonical functional

$$\log \varrho[h|W, \bar{x}] = -\int h(x) \log \frac{h(x)}{w(x;h)} dx - \log \omega, \tag{SI.22}$$

with respect to h under the constraints

$$\int h(x) dx = 1, \quad \int x h(x) dx = \bar{x}. \tag{SI.23}$$

The equivalent unconstrained maximization is

$$\max_h \left\{ -\int h(x) \log \frac{h(x)}{w(x;h)} dx - \log \omega \ -\lambda_0 \left(\int h(x) dx - 1 \right) - \lambda_1 \left(\int x h(x) dx - \bar{x} \right) \right\}, \tag{SI.24}$$

where λ_0 and λ_1 are Lagrange multipliers and $\bar{x}$ and ω are fixed. We set the variational derivative with respect to h equal to zero:

$$0 = -\log h^*(x) - 1 + \log w(x; h^*) - \lambda_0 - \lambda_1 x \tag{SI.25}$$

and solve for h^*:

$$h^*(x) = w(x; h^*) e^{-1-\lambda_0-\lambda_1 x} \tag{SI.26}$$

Setting $q = e^{1+\lambda_0}$, $\beta = \lambda_1$ we obtain

$$\boxed{h^*(x) = w(x; h^*) \frac{e^{-\beta x}}{q}.} \tag{SI.27}$$

This is the same as the most probable distribution in the canonical space.

Relationships for $\log \omega$ *(Equations 29 and 31)*

We write the microcanonical probability functional in the equivalent form

$$\log \varrho[h|W, \bar{x}] = -\log h(x) \log h(x) dx + \int h(x) \log w(x; h) - \log \omega. \tag{SI.28}$$

With Eq. (SI.1) for $\log W[h]$ this becomes

$$\log \varrho[h|W, \bar{x}] = S[h] + \log W[h] - \log \omega, \tag{SI.29}$$

where

$$S[h] = -\int h(x) \log h(x) dx. \tag{SI.30}$$

Applying the condition $\varrho[h^*|W, \bar{x}] = 1$ we obtain

$$\boxed{\log \omega = S[h^*] + \log W[h^*],} \tag{SI.31}$$

which is Eq. (29) in the text.

The entropy of the most probable distribution is

$$\begin{aligned} S[h^*] = -\int h^*(x) \log \left(w(x; h^*) \frac{e^{-\beta x}}{q} \right) dx \\ = -\int h^*(x) \log w(x; h^*) dx + \int (x + \log q) h^*(x) dx \\ = -\log W[h^*] + \beta \bar{x} + \log q. \end{aligned} \tag{SI.32}$$

We substitute this result into Eq. (SI.31) to obtain

$$\boxed{\log \omega = \beta \bar{x} + \log q.} \tag{SI.33}$$

This is Eq. (31) in the text.

Curvature of $\log\omega$ (Equation 33)

Here we show that $\log\omega$ is concave function of $\bar{x}$. Consider the microcanonical spaces of distributions with means $\bar{x}_1$ and $\bar{x}_2$ and let h_1^* and h_2^* be the most probable distributions in these spaces. We form the distribution h by linear combination of h_1^* and h_2^*,

$$h = \alpha h_1^* + (1-\alpha)h_2^*, \quad (0 \le \alpha \le 1), \tag{SI.34}$$

whose mean is $\bar{x} = \alpha\bar{x}_1 + (1-\alpha)\bar{x}_2$. Let h^* be the most probable distribution in the space of distributions with mean $\bar{x}$. We then have:

$$\log\omega(\bar{x}) = \log\varrho[h^*|W,\bar{x}] \ge \log\varrho[\alpha h_1^* + (1-\alpha)h_2^*|W,\bar{x}] \tag{SI.35a}$$

$$\ge \log\varrho[\alpha h_1^*|W,\bar{x}_1] + \log\varrho[(1-\alpha)h_2^*|W,\bar{x}_2] \tag{SI.35b}$$

$$\ge \alpha\log\varrho[h_1^*|W,\bar{x}_1] + (1-\alpha)\log\varrho[h_2^*|W,\bar{x}_2] \tag{SI.35c}$$

$$= \alpha\log\omega(\bar{x}_1) + (1-\alpha)\log\omega(\bar{x}_2). \tag{SI.35d}$$

Here Eq. (SI.35a) expresses the microcanonical inequality in the ensemble $(h;\bar{x})$; Eq. (SI.35b) expresses the concave property of $\log\varrho$; Eq. (SI.35c) expresses the homogeneity of $\log\varrho$; Eq. (SI.35d) expresses Eq. (SI.31) in microcanonical ensembles $(h_1;\bar{x}_1)$ and $(h_2;\bar{x}_2)$. The final result is

$$\log\omega(\alpha\bar{x}_1 + (1-\alpha)\bar{x}_2) \ge \alpha\log\omega(\bar{x}_1) + (1-\alpha)\log\omega(\bar{x}_2) \tag{SI.36}$$

and states that $\log\omega(\bar{x})$ is a concave function of $\bar{x}$. It follows that

$$\boxed{\frac{\partial^2\log\omega}{\partial\bar{x}^2} \le 0,} \tag{SI.37}$$

which is Eq. (33) in the text.

Existence of W (Equation 41)

Given the functional derivative

$$\log w(x) = \log f(x) + a_0 + a_1 x, \tag{SI.38}$$

the selection functional is obtained via the Euler theorem

$$\log W[h] = \int_0^\infty h(x)\log w(x)dx = \int_0^\infty h(x)\log f(x)dx + a_0 + a_1\bar{x} \tag{SI.39}$$

and the functional on the left-hand side of Eq. (34) becomes

$$J[h] = -\int_0^\infty h(x)\frac{h(x)}{f(x)}dx + a_0 + a_1\bar{x}. \tag{SI.40}$$

This is maximized by $h = f$ (a_0, a_1 and $\bar{x}$ are constant) and its maximum is $J[f] = a_0 + a_1\bar{x}$. We set

$$q = \log a_0, \quad \beta = a_1, \quad \log\omega = a_0 + a_1\bar{x}, \tag{SI.41}$$

then using Eq. (SI.38) along with Eq. (42) we note that Eqs. (35), (36), (37) and (39) are all satisfied. The selection functional in Eq. (40) is a special case of (SI.38) with $a_0 = a_1 = 0$, therefore it also satisfies the theorem.

Entropic selection functional Eq. (45)

First we write the entropy functional in the homogeneous form

$$S[h] = -\int_0^\infty h(x)\log\frac{h(x)}{\mu_0[h]}dx, \tag{SI.42}$$

where $\mu_0[h]$ is the zeroth order moment of h:

$$\mu_0[h] = \int h(x)dx. \tag{SI.43}$$

This entropy functional is homogeneous in h with degree 1 and reverts to the Shannon/Gibbs entropy functional when h is normalized to unit area. The functional derivative of the homogeneous entropy functional is

$$\frac{\delta S[h]}{\delta h} = -\log\frac{h(x)}{\mu_0[h]} \tag{SI.44}$$

and satisfies the Euler theorem,

$$S[h] = \int_0^\infty h(x)\left(\frac{\delta S[h]}{\delta h}\right)dx. \tag{SI.45}$$

The entropic selection functional is $\log W[h] = S[h]$ and its functional derivative for h normalized to unit area is $\log w(x) = -\log f(x)$ from which we obtain

$$w(x) = 1/f(x). \tag{SI.46}$$

We insert this into Eq. (47),

$$f(x) = \frac{1}{f(x)}\frac{e^{-\beta x}}{q}, \tag{SI.47}$$

and solve for $f(x)$:

$$f(x) = \frac{e^{-\beta x/2}}{\sqrt{q}}. \tag{SI.48}$$

We obtain the parameters β and q from the zeroth and first order moments:

$$1 = \int_0^\infty \frac{e^{-\beta x/2}}{\sqrt{q}} dx = \frac{2}{\beta\sqrt{q}} \tag{SI.49}$$

$$\bar{x} = \int_0^\infty x \frac{e^{-\beta x/2}}{\sqrt{q}} dx = \frac{4}{\beta\sqrt{q}} \tag{SI.50}$$

We find

$$\beta = 2/\bar{x}, \quad q = \bar{x}^2. \tag{SI.51}$$

In combination with (SI.46) and (SI.48) we obtain w in the form

$$w(x) = \bar{x} e^{x/\bar{x}}. \tag{SI.52}$$

The microcanonical partition function is

$$\log \omega = \bar{x}\beta + \log q = 2 + 2\log \bar{x}. \tag{SI.53}$$

Equations (SI.51), (SI.52) and (SI.53) summarize the results of the entropic selection functional.
